# A preclinical model for identifying rats at risk of alcohol use disorder

**DOI:** 10.1038/s41598-017-09801-1

**Published:** 2017-08-25

**Authors:** Kshitij S. Jadhav, Pierre J. Magistretti, Olivier Halfon, Marc Augsburger, Benjamin Boutrel

**Affiliations:** 1 Center for Psychiatric Neuroscience, Department of Psychiatry, Lausanne University Hospital, Lausanne, Switzerland; 2 King Abdullah University of Science and Technology (KAUST), Thuwal, Saudi Arabia; 3 Brain Mind Institute, Ecole Polytechnique Fédérale de Lausanne (EPFL), Lausanne, Switzerland; 4 Division of Adolescent and Child Psychiatry, Department of Psychiatry, Lausanne University Hospital, Lausanne, Switzerland; 5 Toxicology and Forensic Chemistry Unit, University Center of Legal Medicine, Lausanne, Geneva Switzerland

## Abstract

Alcohol use is one of the world’s leading causes of death and disease, although only a small proportion of individuals develop persistent alcohol use disorder (AUD). The identification of vulnerable individuals prior to their chronic intoxication remains of highest importance. We propose here to adapt current methodologies for identifying rats at risk of losing control over alcohol intake by modeling diagnostic criteria for AUD: inability to abstain during a signaled period of reward unavailability, increased motivation assessed in a progressive effortful task and persistent alcohol intake despite aversive foot shocks. Factor analysis showed that these three addiction criteria loaded on one underlying construct indicating that they represent a latent construct of addiction trait. Further, not only vulnerable rats displayed higher ethanol consumption, and higher preference for ethanol over sweetened solutions, but they also exhibited pre-existing higher anxiety as compared to resilient rats. In conclusion, the present preclinical model confirms that development of an addiction trait not only requires prolonged exposure to alcohol, but also depends on endophenotype like anxiety that predispose a minority of individuals to lose control over alcohol consumption.

## Introduction

Alcohol use disorder, which includes the spectrum of drinking behaviors and consequences ranging from risky use to heavy dependence, has been linked to a wide array of health and social problems^1^. Unhealthy alcohol use accounts for an estimated 85,000 deaths at an economic cost of nearly $250 billion annually in the United States^2^. In 2012, approximately 3.3 million deaths worldwide, representing 5.9% of all global deaths, and 139 million disability adjusted life years (5.1% of the global disease burden) were attributed to alcohol use^3^. At the clinical level, alcohol use disorder, defined as an excessive ethanol seeking and taking with continued consumption despite harmful consequences^4^, is associated with the occurrence of around 200 co-morbid diseases, the most notable amongst them being cardiovascular diseases, gastrointestinal diseases, various cancers and neuropsychiatric disorders^3, 5, 6^. Meanwhile, risk drinking, defined as an average of 15 or more standard drinks per week, or 5 or more on an occasion for men (8 and 4, respectively for women) can be clinically silent yet have adverse health and social consequences^7^. This clinical observation emphasizes the need to identify other criteria than abuse or dependence to better capture the mild to intermediate severity range of the disorder^8^.

Preclinical models of excessive alcohol intake have long been studied, not only for testing and developing effective drugs to alleviate signs of alcohol addiction, but also for understanding the neurobiological substrates of alcoholism^9^. Many strategies used the knockout of one specific gene^10, 11^, or studied alcohol-preferring strains^12, 13^, and used continuous or intermittent access to alcohol to address behavioral responses to alcohol intoxication^14–16^. With one core symptom of drug addiction being the high risk of relapse after a period of abstinence, the extinction/reinstatement model has long been used for assessing the neurobiological mechanisms underlying drug seeking behaviors^17–19^ with the aim of screening effective drug treatments to prevent relapsing behaviors. One limitation of this approach is the lack of consideration for individual vulnerability since all subjects are treated with similar drug doses to assess the pharmacological impact of those drugs to prevent cue-, stress- or priming-induced reinstatement of drug seeking behaviors^20^. Ethanol vapor model has been used for several decades for inducing ethanol dependence^21^. Although the model has good predictive validity in deciphering mechanisms and potential treatments for the human condition, it has rather poor face validity because animals are intoxicated independently of their will^22^. While most studies still defend pharmacology-centered views and models that do not really capture the inter-individual vulnerability to lose control over alcohol consumption^20^, a few studies depict the behavioral traits predicting alcohol drinking in outbred rats^16, 23, 24^, among which the impact of social^25–27^ or traumatic stress^28^ have been shown to promote excessive alcohol drinking.

Overall, the scientific literature reports tremendous amounts of publications deciphering the neurobiological mechanisms underpinning the brain adaptations responsible for alcohol dependence. However, the triggering mechanisms by which a healthy individual, prone to become alcoholic, slowly loses control over alcohol consumption remains to be fully elucidated^29^. The inability to control drug taking in general and conditioned responses in particular, is a complex brain disorder that affects the most vulnerable individuals and worsens with recurring drug consumption. Further, understanding the heterogeneity in the behavioral characteristic of patients with alcohol use disorders is warranted for developing personalized treatments^30^, notably in the light of the overall modest effect size for approved and experimental pharmacotherapies^31^.

Considering that treatments, on an average, produce limited improvements in ‘alcoholics’, one could expect considerable clinical benefits in categorizing subpopulations of patients according to their biology^32^. Because alcohol misuse is a complex neurobiological disorder, investigations based on one preclinical model operationalizing one diagnostic criterion are rather challenging. Among the number of unresolved methodological and conceptual issues that need to be carefully considered and addressed to advance the field, neuroscience-based clinical phenotypes could advance genetic studies of alcoholism etiology and treatment. Over the last decade, converging evidence demonstrated that preclinical research could successfully integrate several dimensions of addiction following the Diagnostic and Statistical Manual^33^. This research has identified specific phenotypes and markers that underlie individual vulnerability to develop addiction-like behaviors in rats consuming cocaine^34^. Not only this approach revealed a large spectrum of severity for cocaine abuse in rats, but it also allowed to better depict the neurobiological adaptations specifically occurring in the brain of rats developing the behavioral hallmarks of cocaine addiction^35–42^.

The recently revised DSM5 criteria defines alcohol misuse as a single disorder, with a broad spectrum from mild to severe conditions, whereas former DSM-IV emphasized the distinction between alcohol abuse and alcohol dependence. The current consensus acknowledges that DSM5 criteria advantageously represent the clinical experience^33, 43^. Taking cue from past observations inspired by clinical experience^34^, we report here a novel animal model addressing alcohol use disorder. In brief, three criteria of the DSM have been operationalized in rats: (1) inability to cease drug seeking during signaled unavailability, (2) higher motivation for drug intake, and (3) continued drug use despite adverse consequences. Rats identified as positive for all these criteria, defined as vulnerable, developed uncontrolled conditioned responses and enhanced alcohol use as compared to rats failing to meet any of these criteria, accordingly named resilient.

## Results

**Figure 1:**
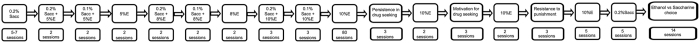
Flowchart for operant training for ethanol self-administration (Sacc: Saccharine concentration expressed as percentage in weight/volume, E: Ethanol concentration expressed as percentage in weight/volume).

**Figure 2:**
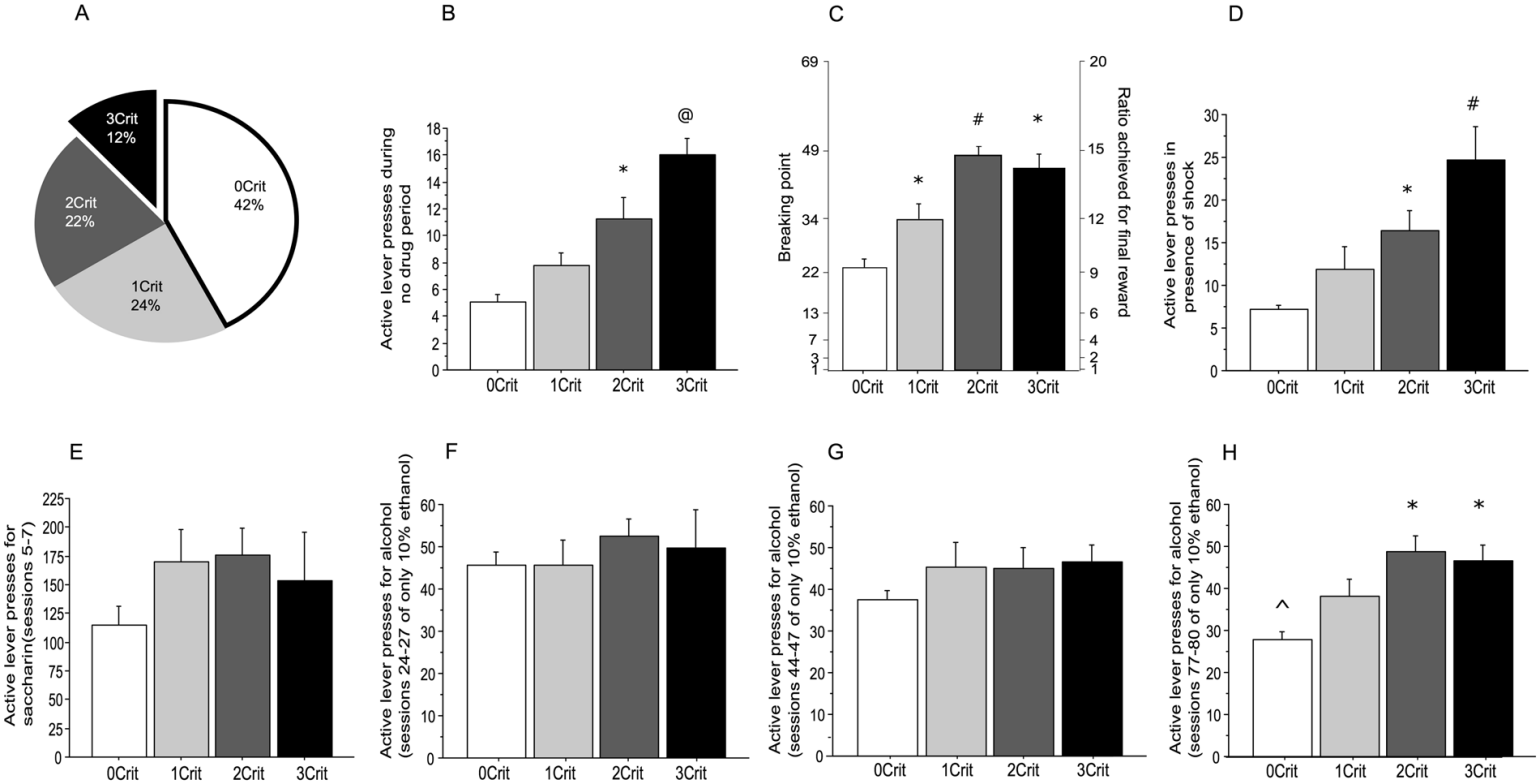
Identification of Addiction-like behaviors in rats. (**A**) Percentage of the total population (n = 59) of rats positive for zero (0Crit), one (1Crit), two (2Crit), or three addiction-like criteria (3Crit). (**B**) Persistence in drug seeking, as measured by number of lever presses (±SE) during the no-drug period. (**C**) Motivation for the drug, as measured by the breaking point (±SE) during a progressive-ratio schedule of reinforcement. (**D**) Resistance to punishment, as measured by the number of lever presses (±SE) when alcohol delivery was associated with a mild electric foot shock (0.22 mA). (**E**) Average number of lever presses (±SE) for saccharine 0.2% (sessions 5–7). (**F**) Average number of lever presses (±SE) for ethanol 10% (sessions 24–27). (**G**) Average number of lever presses (±SE) for ethanol 10% (sessions 44–47). (**H**) Average number of lever presses (±SE) for ethanol 10% (sessions 77–80). ^*^Significant as compared to 0crit, ^#^Significant as compared to 0crit and 1crit, and ^@^Significant as compared to 0crit, 1crit and 2crit, ^Significant as compared to 0crit rats during session 24–27.

At the end of the baseline self-administration period (80 sessions during which animals were trained under a fixed ratio 1, time out 4 sec to get 0.1 mL of 10% w/v ethanol, see Fig. 1), rats underwent a procedure for screening evidence for addiction-like behavior^35^. A rat was considered positive for an addiction-like criterion when its score was in the 66th to 99th percentile of the distribution. Hence, of the total 59 rats, we obtained 4 groups, 25 rats with 0 criterion (42.4%), 14 rats with 1 criterion (23.7%), 13 rats with 2 criteria (22%) and 7 rats with 3 criteria (11.9%) (Fig. 2A).

### Persistence in lever pressing during the no-drug period

The mean number of lever presses for each group were 5.07 ± 0.55 (0crit), 7.78 ± 0.92 (1crit), 11.28 ± 1.6 (2crit) and 16.04 ± 1.17 (3crit), respectively (Fig. 2B). A one-way ANOVA showed a significant difference between groups (F_3,55_ = 18.345, *p* < 0.0001). Post hoc Tukey’s tests revealed that, during the no-drug period, the 3crit rats exhibited higher lever presses compared to all the other groups (vs 0crit p < 0.0001, vs 1crit p < 0.001, vs 2crit p < 0.048). The 2 criteria rats differed from 0 criteria rats (p < 0.001) but were comparable to 1 criteria rats (p = 0.092). The 0 and 1 criterion rats had similar performances (p = 0.157).

### Increased motivation for alcohol seeking and drinking in an effortful condition

The respective breaking points were 23.17 ± 1.76 (0crit), 34.19 ± 3.65 (1crit), 48.2 ± 2.24 (2crit) and 46.09 ± 2.90 (3crit) (Fig. 2C). One-way ANOVA showed a statistically significant difference among the groups (F_3,55_ = 22.217, *p* < 0.0001). Post hoc Tukey’s tests revealed that the 3crit rats displayed an increased motivation for ethanol seeking compared to 0crit rats (p < 0.0001). Despite a large difference, the breaking point displayed by 3crit rats failed to be statistically different from that of 1crit animals (p = 0.058), while 2crit and 3crit rats exhibited similar performances (p = 0.96). The 2crit rats displayed a higher breaking point compared to 0crit and 1crit rats (vs 0crit p < 0.001, vs 1 crit p = 0.003). Finally, even the 1crit rats exhibited a higher breaking point compared to the 0crit rats (p = 0.008).

### Resistance to punishment

The mean number of lever presses for each group were 7.17 ± 0.5 (0crit), 11.93 ± 2.53 (1crit), 16.44 ± 2.33 (2crit) and 24.62 ± 3.99 (3crit rats) (Fig. 2D). One-way ANOVA showed a statistically significant difference among the groups (F_3,55_ = 12.53, *p* < 0.0001). Post hoc Tukey’s tests indicated that 2 and 3crit rats had similar resistance to punishment (p = 0.084) but 3crit rats accepted more shocks than 0crit (p < 0.0001) and 1crit rats (p = 0.002). Whereas 2crit rats were not different from 1crit rats (p = 0.372), they exhibited a clear-cut resistance to punishment as compared to the 0crit rats (p = 0.002). Finally, the 0crit and 1crit rats manifested similar performance in resisting to punishment (p = 0.207).

Once the four groups of rats were identified, we analyzed *a posteriori* individual performance during the baseline conditioning to address the evolution of alcohol seeking and taking over the course of the instrumental conditioning (Fig. 2E,F,G,H). A Pearson’s Correlation analysis was done between the three addiction-like behaviors, the active lever presses towards the end of 0.2% saccharine training (when rats displayed the highest lever pressing activity, Fig. 2E) and at three different time points of the ethanol operant conditioning (Fig. 2F,G,H). Remarkably, the three criteria scores were all positively correlated with the average active lever presses at the end of ethanol training only (session 77–80) (Table 1). It is important to note that whereas most of the rats maintained constant lever pressing behavior, 0-crit rats displayed reduced conditioned responses over time, reflecting a growing disinterest for lever pressing (Fig. 2 and Supplementary Figure 1). Rats exhibited similar weight gain during the 80 sessions of ethanol intake (Supplementary Figure 2A and B), which excludes a body size-induced bias in alcohol consumption. Interestingly, while all rats exhibited similar (and rather high) levels of ethanol intake after 25 sessions of ethanol operant conditioning (around 2 g/kg within 30 min), 2- and 3-crit rats consumed 1.5 times as much amount of alcohol after 80 sessions of ethanol self-administration (roughly 0.9 versus 0.6 g/kg, Supplementary Figure 2C and D), confirming that the development of excessive conditioned responses to alcohol requires a prolonged exposure to alcohol context.

**Table 1:** Correlational analysis between the three addiction-like criteria and active lever presses measured during 4 key phases of the operant conditioning.

	Average active lever presses for saccharine only (session 5–7)	Average active lever presses for ethanol only (session 24–27)	Average active lever presses for ethanol only (session 44–47)	Average active lever presses for ethanol only (session 77–80)
Persistence in drug seeking during the no-drug period	−0.180	0.267	0.208	0.378*
Excessive motivation for alcohol seeking and drinking	0.25	0.227	0.385*	0.649*
Resistance to punishment	0.373*	−0.055	0.102	0.362*

*p < 0.05 Significant using Pearson’s correlational analysis (2 tailed).

### Addiction score

**Figure 3:**
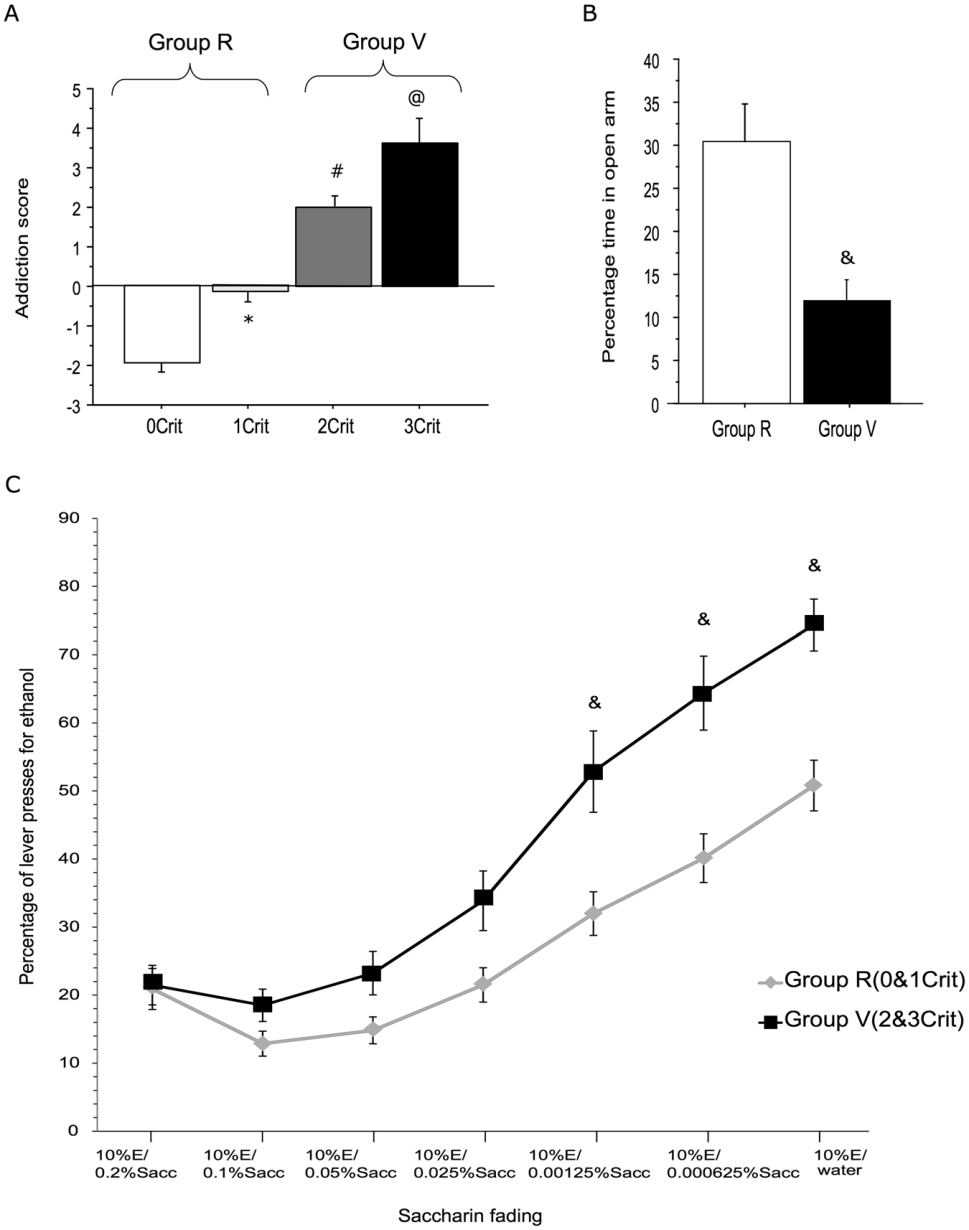
Identification of addiction-vulnerable and addiction-resilient rats. (**A**) The four experimental groups were linearly ranked along the addiction score (±SE), with negative addiction scores for 0crit and 1crit rats, and positive addictions scores for 2crit and 3crit rats, identifying two groups of rats, the addiction resilient (Group R, n = 39) and the addiction vulnerable (Group V, n = 20). (**B**) Anxiety-related behavior, as measured by the percentage of time (±SE) spent in the open arms during a 5-min exposure to an elevated plus-maze in resilient (n = 35) and vulnerable (n = 16) animals. (**C**) Comparison of alcohol versus saccharine consumption in a two-choice paradigm. Data represent the number of lever presses for ethanol 10% in percent of total lever presses (ethanol + saccharine). The concentration of saccharine solution was reduced (0.2 0.1, 0.05. 0.025, 0.0125, 0.0062, 0%w/v) until rats were given the choice between alcohol and tap water (Group R: n = 32, Group V: n = 10). ^*^Significant as compared to 0crit, ^#^Significant as compared to 0crit and 1crit, and ^@^Significant as compared to 0crit, 1crit and 2crit, ^&^Significant as compared to group R.

The addiction score was calculated as the sum of the standardized scores of each of the addiction-like criteria^44^. The addiction scores were −1.96 ± 0.2, −0.15 ± 0.25, 1.98 ± 0.26 and 3.64 ± 0.6, respectively (Fig. 3A). A one-way ANOVA demonstrated a significant main effect for addiction score among the groups (F_3,55_ = 71.33, p < 0.0001). Not only Tukey’s post-hoc tests revealed that all addiction scores statistically differed one from each other, but a Pearson’s analysis revealed a positive correlation between the three addiction-like criteria and the addiction score confirming that the addiction score is highly representative of the three addiction-like criteria (Persistence in drug seeking during the no-drug period: r = 0.794; p < 0.001, Excessive motivation for alcohol seeking: r = 0.818; p < 0.001, Resistance to punishment: r = 0.641; p < 0.001).

Strikingly, the addiction score also correlated positively with active lever presses for ethanol towards the middle (session 44–47) (Pearson’s correlation: r = 0.305, p = 0.018) and end of the training (session 77–80) (Pearson’s correlation: r = 0.609, p < 0.0001). But it did not correlate with active lever presses for saccharine or lever presses for ethanol at the beginning of the training (session 24–27).

### Factor analysis

A factor analysis was conducted for the three addiction-like criteria to determine whether they loaded on the same underlying construct. The eigenvalue was kept as 1. Remarkably, all the three addiction-like criteria loaded on one factor only (Persistence in drug seeking during the no-drug period: r = 0.819, Excessive motivation for alcohol seeking: r = 0.818, Resistance to punishment: r = 0.641) accounting for 58% of the variance, further supporting that the three addiction-like criteria are measures of a single underlying factor that represents persistent/compulsive drug use.

As can be seen above, 0crit and 1crit rats are rather comparable to each other, with negative addiction scores, while 2crit and 3crit are quite similar, with both displaying positive addiction scores. Hence, we clubbed together the data of criteria 0 and 1 rats (addiction trait negative or resilient = Group R) and the data of criteria 2 and 3 rats (addiction trait positive or vulnerable = Group V).

### Comparison of anxiety trait

To evaluate whether anxiety-like behavior could represent a predictive trait for future addictive-like behavior, we analyzed *a posteriori* individual performance on the elevated plus maze, measured right after the end of the saccharine fading procedure. The percent time spent in the open arm for Resilient and Vulnerable rats was 30.36 ± 4.58 and 11.99 ± 2.34, respectively. A series of 8 rats observed on the same day was lost due to technical failure. An unpaired T test showed that the Vulnerable rats spent significantly lesser time in the open arm (t(49) = 2.65, p = 0.0106), suggesting an anxious profile, most likely representing a predisposing characteristic for developing alcohol use disorder in rats (Fig. 3B).

### Evaluation of alcohol consumption vs. saccharine fading in a two-choice paradigm

The rats were provided a choice between 10% w/v ethanol and fading concentrations of saccharine starting from 0.2% w/v saccharin to finally water. A two-way repeated measures ANOVA was conducted with the two groups as the between subject factor, and the ethanol/saccharine fading procedure as the within subject factor. The analysis revealed a significant group effect (F_8,48_ = 4.047, p = 0.001), a significant effect of the choice procedure (F_6,48_ = 93.77, p < 0.0001) and a significant effect of the group x choice interaction (F_186,48_=1.61, p=0.0267). Post hoc analyses revealed that the Vulnerable rats significantly preferred ethanol compared to Resilient ones for saccharine concentrations lower than 0.0125% (Fig. 3C).

Finally, confirming that the addiction score is highly representative, a Pearson’s correlation analysis showed a linear positive correlation between the addiction score and 10% w/v ethanol vs water choice, which was statistically significant (r = 0.331, p = 0.037).

### Evaluation of blood alcohol levels elimination in vulnerable versus resilient rats

**Figure 4:**
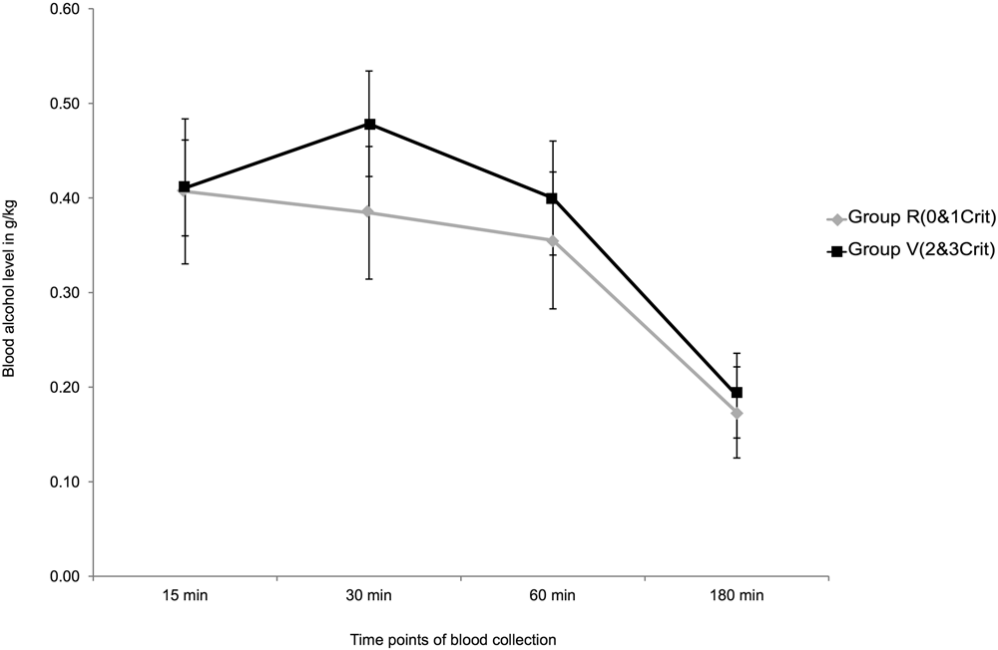
Ethanol elimination rates measured in resilient (Group R, n = 12) and vulnerable (Group V, n = 7) animals 15, 30, 60 and 180 min after ethanol administration by oral gavage (1 g/kg). Data represent blood alcohol levels (±SE) expressed in g/kg.

In a last set of experiments, we measured blood alcohol levels (BAL) in a subset of vulnerable and resilient rats, at 15, 30, 60 and 180 min after ethanol gavage (1 g/kg). A two-way repeated measures ANOVA revealed a significant decrease in BAL over time (F_3,57_ = 24, p < 0.0001), but no difference between groups (F_1,17_ = 0.2, p = 0.66) (Fig. 4).

## Discussion

In 1990, H.A. Skinner emphasized that “Most adults in North America are either light drinkers or abstainers, so alcohol does not cause them problems. However, a small but often highly visible minority - approximately 5% of the adult population - show major symptoms of alcohol dependence. Between these extremes, there is a sizable group of about 20% of the population, particularly young men, who are drinking at risk levels and have encountered some problems related to their alcohol use”^45^. Later, R. Saitz confirmed this observation and underscored that “The prevalence of unhealthy use is 7 to 20 percent or more among outpatients, 30 to 40 percent among patients in emergency departments, and 50 percent among patients with trauma”^1^. These two observations highlighted the broad spectrum of alcohol consumption levels, and posed the question about “problem drinkers” in the general population who are not manifesting major symptoms of alcohol dependence but are drinking at levels that increase risks for medical and psychosocial consequences^45^.

Understanding aspects of addiction neuroscience requires appropriate preclinical models that should be highly representative of the clinical understanding of addiction disease. Most of the animal models used for the past decades have resulted in incremental growth in understanding the pharmacological basis of drug intoxication^46–50^. However, the construct of drug self-administration is different from that of drug addiction, simply because most of the recreational drug users keep control and do not encounter any significant problem. While it has long been thought that repeated exposure to drugs of abuse should lead to addiction-like phenotypes^48, 50, 51^, converging evidences now point out to individual vulnerabilities towards developing addiction-like state^34, 52^. In line with these recent reports, our preclinical data extend clinical findings showing that addiction-like trait development not only requires prolonged exposure to alcohol but also depends on individual vulnerabilities.

By adapting a preclinical model implementing the operational definitions used in the DSM criteria^35^, we focused on an individualized approach which is of paramount importance considering that all individuals have varied risks of developing addiction. The three addiction-like criteria (see supplementary information for further discussion on data selection) can be considered as representatives of different items in DSM 5; persistent or uncontrolled alcohol seeking (item 2), excessive motivation (item 3 and 4), and resistance to adverse consequences (item 6, 7, 8 and 9). We performed a factor analysis to assess whether one factor could explain the variety of results on these 3 different tests; in other words, we verified whether the variables were inter-related. Strikingly, we demonstrated that the three addiction-like criteria loaded on one factor only, revealing that addiction-like criteria are measures of a single underlying/latent construct which we could consider being perseverative/compulsive drug use. It could also refer to the concept of “loss of control-prone phenotype”^44^ although the neurobiological and psychological underpinnings of this deserves further studies to be confirmed in this model. It is important to stress out that our procedure aims at identifying rats with excessive conditioned responses, and we pose the question of knowing whether these rats are at risk of losing control over alcohol intake. Although all rats exhibited rather high levels of ethanol consumption initially (around 2 g/kg shortly after the end of the saccharine fading procedure), they stabilized their intake in the range of 0–6 to 0.9 g/kg afterwards. Importantly, the operant conditioning lasted for more than 3 months, but daily sessions were limited to 30 min only, which is not sufficient for triggering a binge drinking behavior that brings blood alcohol levels to 80 mg/dl and above^15, 53^. Our working hypothesis did not aim at evaluating the development of addiction criteria over time. Consequently, we only measured the addiction-like behaviors after 3 months of operant conditioning considering that, according to a recent report, intermittent access to alcohol led to the development of quinine-resistant intake after 3 months only^54^. Although we have not tested continued intake despite adulteration of alcohol with the bitter tastant quinine, we make the reasonable assumption, although it as to be tested, that our vulnerable rats would display quinine-resistant alcohol intake, in contrast to resilient rats, given recent evidence suggesting the similarities between quinine- and footshock-resistant alcohol consumption^24, 55^.

One criticism on this model could raise the question about a potential selection bias given that all criteria are associated with lever pressing behavior. In this perspective, the underlying construct measured here would not be nothing more than lever pressing capacity. In other words, rats that are inherently good at pressing the lever would be selected, and these three parameters computed by us would not truly model addiction-like trait, but motor performance. Therefore, we conducted a correlation analysis between the three addiction-like criteria and active lever presses measured during 4 key phases of the operant conditioning. Remarkably, whereas rats’ behavior during saccharin training or during the initial sessions of alcohol conditioning could not predict future addiction score, the Pearson’s correlation analysis clearly demonstrated that higher alcohol responses predicted the severity of the addiction score after a long history of alcohol consumption only. It is important to note that only 0-crit rats (the larger proportion of the sample) displayed reduced conditioned responses over time, reflecting a growing disinterest for lever pressing that is not surprising with aging.

The addiction score calculated as the sum of the standardized scores of each of the addiction-like criteria in our rats resembles the addiction severity index (ASI) in humans^56^. The scores for each group were significantly different from each other, and were linearly increasing from 0crit to 3crit rats. Noteworthy, the scores of 0crit and 1crit rats were negative and those of 2crit and 3crit rats were positive, further supporting our claim of clubbing them together and naming them addiction trait negative (addiction resilient) and addiction trait positive (addiction vulnerable), respectively.

Again, because one possible confounding interpretation on rats’ ability to perform highly during the screening for addiction-like criteria could have been the long lasting operant conditioning, with rats exhibiting higher levers pressing capacities ultimately displaying the highest addiction scores, rats were given a choice between 10% w/v ethanol and decreasing concentrations of saccharine. The addiction vulnerable group showed a higher preference for alcohol as compared to the addiction resilient group. In this perspective, whatever the amount of effort displayed by rats, resilient animals never exhibited a significant preference for the alcohol lever, whereas vulnerable rats started displaying a preference for ethanol with the lowest saccharine concentrations. This further strengthens the discriminating ability of our model for identifying rats having higher propensity to develop addiction-like traits.

We also analyzed whether the anxiety trait tested at the beginning of the experiment was observed in rats with increased propensity to develop addiction-like traits. There was a higher incidence of anxiety trait in the vulnerable group as compared to the resilient one. Not only we confirm that high anxiety plays an important role in ethanol self-administration^34, 57^, but we also corroborate human epidemiological studies showing that anxiety and risk of alcohol abuse are co-morbid conditions^58^.

Finally, we have shown that both groups of rats have similar alcohol elimination rates, suggesting that most likely, this animal model of vulnerability to lose control over alcohol consumption does not depend on the pharmacokinetic of ethanol and its metabolism.

However, the current set of experiments present a few limitations that need to be addressed soon. First, we claim that our model most likely identifies animals at risk of losing control over alcohol consumption, possibly triggering escalated intake over time. Therefore, it is important to measure alcohol intake in vulnerable and resilient rats following continuous and intermittent alcohol access in home cages^14, 16, 24, 53^. Second, with impulsivity being demonstrated to predict a switch to compulsive drug and palatable food taking^36, 59^, assessing whether impulsive trait correlates with the alcohol addiction criteria is critical. Third, assessing whether the vulnerability to relapse correlates with the alcohol addiction criteria is a necessary step for confirming the face validity of our model. Finally, evaluating the effects of medication treatments known to reduce alcohol craving in each group is compulsory for addressing the predictive validity of this model.

In conclusion, we have collected a series of observations demonstrating that only a minority of rats would exhibit an addiction-like behavior for alcohol. By adapting to rodents a few criteria used for screening drug addiction according to the DSM^35^, we contribute to recognize that “addiction is a progressive disorder and to some extent iodiosyncrasic”^34^. Since the success rate of current treatments for alcohol use disorders is at best average, we consider this endeavor is an original development of current methodologies, allowing preclinical studies to investigate biobehavioral predispositions to lose control over alcohol consumption using a singular translational approach.

## Materials and Methods

Further details are provided in Supplementary Information.

### Animals

Male Wistar rats (n = 59) were bred in-house in the institute’s animal facility (breeders ordered from Charles River, France), they were approximately 7 weeks old and weighed 200–250 grams at the beginning of the experiment. They were kept in reversed 12-h light/dark cycle (lights off at 8.30 am) and housed in controlled temperature and humidity conditions. All experiments were conducted in accordance with the Swiss National Institutional Guidelines on Animal Experimentation, and approved by the Swiss Cantonal Veterinary Office Committee for Animal Experimentation (#1999 to Dr. Benjamin Boutrel).

### Screening for addiction-like behavior

Between test sessions aiming at scoring addiction-like behaviors (3 daily consecutive sessions each time, Supplementary Figure 3), rats underwent 2 consecutive sessions of basic training during which they were trained again under the same baseline conditions.

A) **Inability to abstain during a signaled period of reward unavailability**
  Rats underwent 3 daily consecutive sessions; each one consisted of an 8 minute-period of reward availability, followed by a 4 minute-period of signaled unavailability. Repeated three times, this sequence resulted in a total of 36 min. The period of unavailability was signaled by lighting up the self-administration chamber house light and interrupting the cubicle fan. The light diode above the active lever remained off after lever presses, and alcohol was not delivered. The average number of active lever presses during the signaled unavailability periods indicated the persistence in drug seeking during the no drug period.
B) **High motivation for alcohol seeking**
  Rats were required to progressively increase the number of active lever presses between two successive rewards based on the progression sequence given by the following formula: response ratio = (5e^(reward× 0.2)^) − 5^60^. Hence, the progressive-ratio schedule followed the progression: 1, 2, 4, 6, 9, 12, 15, 20, 25, 32, 40, 50, 62, 77, 95, 118, etc. Each session lasted 90 min, or automatically shut down following 20 consecutive minutes of inactivity on the active lever. The maximal number of active lever presses performed to reach the final ratio was defined as the breakpoint, a value reflecting animals’ motivation to get the reward. The breaking point across 3 consecutive daily sessions was averaged and considered as marker of motivation for alcohol seeking.
C) **Resistance to punishment**

In this paradigm, each lever press delivered 0.1 mL of 10% w/v ethanol by means of a dipper, followed by mild electric foot shocks (0.22 mA for 0.5 second) through the grid of the SA chamber when the dipper retracted. This was conducted for 3 consecutive daily sessions, and the average number of active lever presses across these 3 consecutive trials was considered as a marker of resistance to punishment, reflecting a compulsive reward seeking and taking behavior.

### Evaluation of alcohol consumption versus saccharine in a two-choice paradigm

One possible confounding interpretation on rats’ ability to perform highly during the screening for addiction-like criteria could have been the instrumental response to environmental cues, with rats exhibiting higher levers presses during the 80 sessions of ethanol operant conditioning ultimately displaying the highest addiction scores only because of physical capacity to press continuously the active lever. Therefore, rats were given the choice between ethanol 10% and fading concentrations of saccharine (see supplementary information). The concentration of saccharine solution was reduced (0.2 0.1, 0.05. 0.025, 0.0125, 0.00625, 0% w/v) until rats were given the choice between alcohol and tap water. In this last set of experiments, only 42 rats were analyzed, the first 17 rats used in the study were sacrificed before we operationalized these experiments.

### Statistical analysis

Data are expressed as mean ± standard error (SE). Shapiro Wilk test was used to ascertain the normality distribution of the data sets. Parametrical data were analyzed by one way- and two-way ANOVAs followed by Tukey’s tests and Bonferroni correction, respectively. Factor analysis was conducted for the three addiction-like criteria to determine if they loaded on the same underlying construct. Pearson’s correlational analysis was used to determine the correlation between the addiction score, the three addiction parameters, 0.2% saccharine training and ethanol training at different time points of training. Unpaired T test was used to analyze the anxiety data. The level of significance was set at 0.05, and Statistical analyses were performed using IBM SPSS Statistics 23.

## Electronic supplementary material

Supplementary Information
