# Supplementary material for: A preclinical model for identifying rats at risk of alcohol use disorder
Source: Sci Rep. 2017 Aug 25;7:9454. doi: 10.1038/s41598-017-09801-1 (PMC5572732; doi:10.1038/s41598-017-09801-1)

SUPPLEMENTARY INFORMATION FOR

A preclinical model for identifying rats at risk of alcohol use disorder

Kshitij Jadhav[1], Pierre J. Magistretti[1,2,3], Olivier Halfon[4], Marc Augsburger[5], Benjamin Boutrel[1,4]

[1] Center for Psychiatric Neuroscience, Department of Psychiatry, Lausanne University Hospital, Switzerland

[2] King Abdullah University of Science and Technology (KAUST), Thuwal, Saudi Arabia

[3] Brain Mind Institute, Ecole Polytechnique Fédérale de Lausanne (EPFL), Lausanne, Switzerland

[4] Division of Adolescent and Child Psychiatry, Department of Psychiatry, Lausanne University Hospital, Switzerland

[5] Toxicology and Forensic Chemistry Unit, University Center of Legal Medicine, Lausanne - Geneva, Switzerland

Correspondence: Benjamin Boutrel, Laboratory on the Neurobiology of Addictive and Eating Disorders, Center for Psychiatric Neuroscience, Department of Psychiatry, Lausanne University Hospital, Site de Cery, CH-1008 Prilly, Switzerland. Tel: 0041 21 314 9206.

Email: benjamin.boutrel@chuv.ch

This supplementary information contains further material and methods, results and discussion, and Figure S1, Figure S2 and Figure S3.

MATERIAL AND METHODS

Apparatus:

A) Self-administration (SA) chambers

Twelve operant chambers (305 x 241 x 210 mm, Med Associates, St. Albans, Vermont, USA) were used for the experiment. The chambers were housed in larger sound attenuated cubicle, equipped with exhaust fans for air renewal, also used for masking the background noise. The floor was made of a grid capable of delivering electrical shock. Each operant panel contained two retractable levers 60 mm above the grid and 35 mm equidistant from the midline, with a white light diode mounted 30 mm above each lever. Between the two levers was the delivery section which delivered 0.1mL of the fluid by means of a dipper.

B) Elevated plus maze

The arena was positioned 50 cm above the floor, and divided in four arms: two "closed arms" enclosed by plastic wall (500x100x425 mm), and two "open arms" without walls. In the center, a small open arena (100x100 mm) allowed access to each arm. Luminosity was fixed at 20 lux in the open arms, and 5 lux in the closed arms.

Animal's training for alcohol self-administration

Laboratory rodents do not voluntarily consume alcohol to intoxication, in part because of taste aversion. Higher levels of consumption could be achieved by masking the taste of alcohol with saccharine (Roberts *et al.*, 1999), which was faded out as alcohol concentrations increased (Dayas *et al.,* 2007). Rats were trained under a Fixed Ratio 1 - Time Out 4sec schedule of reinforcement for a total of 105, 30-min daily sessions (25 sessions of saccharine fading + 80 sessions of ethanol self-administration). During these baseline conditions, pressing the right (active) lever delivered 0.1 mL of ethanol (10%w/v in tap water, prepared from a 94% (vol/vol) ethanol solution) in the delivery section and

illuminated the diode above the active lever. The left lever was inactive, presses were recorded but had no consequence.

Evaluation of alcohol consumption versus saccharine in a two-choice paradigm

In this set of experiments, between the two levers was the delivery section which delivered 0.1mL of fluids by means of a 2-well metallic drinking cup that allowed for up to 2 solutions to be administered upon the pressing of the appropriate lever. After the last set of experiments aimed at screening addiction-like criteria, rats were trained in this novel environment to press the left lever for accessing saccharine reward (0.1mL of 0.2% w/v saccharine) until they achieved stability in their behavior for two consecutive sessions. They were then exposed to both liquids being available simultaneously, left lever associated with the delivery of saccharine (0.1mL saccharine 0.2%, Sigma-Aldrich, Buchs, Switzerland), and the right lever associated with the delivery of ethanol (0.1mL ethanol 10%w/v). Although the left lever had long been denied (inactive during baseline conditions), rats exhibited a strong preference for this lever now paired with saccharine reward. The concentration of saccharine solution was then reduced (0.2 0.1, 0.05. 0.025, 0.0125, 0.00625, 0 %w/v) until rats were given the choice between alcohol and tap water. This was done to determine whether the vulnerable rats would exhibit higher preference for ethanol as compared to saccharine at different concentrations, independently of their instrumental conditioning (meaning independently of their effortful lever press capacity). The rats were tested twice on each stage and the preference for the alcohol-paired lever (in percent of total lever presses) was averaged for the 2 sessions. Despite a long history of ethanol self-administration (80 sessions), all rats did prefer high doses of saccharine over alcohol 10%, which is not unexpected given that saccharine reinforcing properties are considered higher than those of cocaine itself (Lenoir et al., 2007).

Anxiety measured on elevated plus maze

At the end of the fading procedure, rats were tested in the elevated plus paradigm for evaluating anxiety-like behaviors. The experiment was conducted under a dim light (10-15 Lx), during which animal tracks were recorded for 5 minutes by a digital video camera mounted above the maze and connected to a computer running a tracking-software (Ethovision v.3.1 – Noldus Technology, Wageningen, The Netherlands). The trial was initiated by gently placing the rats in the central area facing one of the open arms. The percentage of time spent in the open arms was considered as a marker of anxiety, with lesser time in open arms pointing towards higher anxiety levels.

Blood sample collection and blood alcohol analysis

The method was described in previous reports (Karbouche *et al.*, 2010). Briefly, blood was drawn from the caudal vein for the determination of ethanol concentrations. Blood samples (200 µL) were collected into 300-µL sodium fluoride tubes (Microvette® 300, Sarstedt, Sevelen, Switzerland) at 15, 30, 60 and 180 min after ethanol administration by oral gavage (1g/kg), and were stored at -20°C until analysis.

Blood ethanol concentrations were determined according to the validated procedure developed in the Toxicology and Forensic Chemistry Unit, at the University Center of Legal Medicine of Lausanne and Geneva. This method used headspace gas chromatography-flame ionization detector using 50 µL of blood and dioxane as internal standard. Each analytical batch included six calibrator samples (from 0.25 to 3 g/kg) and three certified quality control samples (0.49, 0.78, and 1.47 g/kg Medidrug® Ethanol S-plus; Medichem, Steinenbronn, Germany) were analyzed in duplicate. Results obtained for the quality control samples were comprised within the acceptance limits provided by the commercial control. The limit of quantification was 0.05 g/kg.

Statistical analyses

One way ANOVAs with post hoc Tuckey's test was used to analyze addiction-like behaviors and addiction scores. Pearson's correlational analysis was used to determine

the correlation between the addiction score, the three addiction parameters, 0.2% saccharine training and ethanol training at different time points of training. Factor analysis was conducted for the three addiction-like criteria to determine if they loaded on the same underlying construct. A two-way repeated measures ANOVA method was used to analyze the ethanol vs. saccharine procedure and blood alcohol levels elimination. Significant main effects were further analyzed using pair wise comparisons and followed by Bonferroni correction. Statistical analyses were performed using IBM SPSS Statistics 23.

RESULTS

Between test sessions aiming at scoring addiction-like behaviors, rats underwent 2 consecutive sessions of basic training during which they were trained again under the same baseline conditions. The three daily consecutive sessions for each test are presented on Supplementary Figure 3, and the statistical analyses are summarized in the table above.

	**Persistence in lever pressing**	**Motivation for alcohol**	**Resistance to punishment**
**0Crit**	**NS** ($F_{2,48}$=0.16. p=0.85)	**Significant** ($F_{2,48}$=7.13, p=0.001) Session1 significantly higher as compared to Session2 and Session3	**Significant** ($F_{2,48}$=61.93, p<0.0001) Session1 significantly higher as compared to Session2 and Session3
**1Crit**	**Significant** ($F_{2,26}$=4.8, p=0.01) Session1 significantly higher than Session3	**Significant** ($F_{2,26}$=6.26, p=0.006) Session 1 significantly higher as compared to Session2.	**Significant** ($F_{2,26}$=19.15, p<0.0001) Session1 significantly higher as compared to Session2 and Session3
**2Crit**	**Significant** ($F_{2,24}$=4.1, p=0.02) Session 2 significantly higher than Session 1	**Significant** ($F_{2,24}$=4.63, p=0.01) Session 1 significantly higher as compared to Session2 and Session3.	**Significant** ($F_{2,24}$=11.87, p=0.0002) Session 1 significantly higher as compared to Session2 and Session3.
**3Crit**	**NS** ($F_{2,12}$=0.54. p=0.59)	**Significant** ($F_{2,12}$=5.85, p=0.01) Session1 significantly higher than Session3	**NS** ($F_{2,12}$=2.27. p=0.14)

First conclusion, the only experimental condition for which all rats exhibited a reduced lever pressing behaviour along recurrent exposures is the progressive ratio. Second conclusion, 0crit, 1crit and 2crit rats exhibited a reduced lever pressing behaviour with repeated exposure to foot shock, whereas 3crit rats maintained their lever pressing behaviour, hence supporting their compulsive behaviour.

Interestingly, the compulsive criterion does not appear only in the 3Crit rats, and therefore, is not a limitation criterion to addiction trait positive rats: 42% of 1Crit rats (6 out of a total of 14 rats) and 61% of 2Crit rats (8 out of a total of 13 rats) met the inclusion criterion for compulsivity.

	**Active lever presses during no drug period**	**Progressive ratio**	**Resistance to punishment**
**1Crit**	6/14	2/14	6/14
**2Crit**	8/13	11/13	8/13

In the broad spectrum of alcohol drinkers we analyzed, we identified 4 groups of rats, from the most resilient to the most vulnerable ones. The addiction scores for each group were significantly different from each other, and were linearly increasing from 0crit to 3crit rats. The scores of 0crit and 1crit rats were negative and those of 2crit and 3crit rats were positive, therefore supporting our claim of clubbing them together and naming them addiction trait negative (addiction resilient, R) and addiction trait positive (addiction vulnerable, V), respectively. Addiction trait negative group had 39 rats (66.1%) and Addiction trait positive group had 20 rats (33.9%), and it seemed relevant to compare their respective behavior along key phases of the operant conditioning procedure.

Comparison of saccharine lever presses at the beginning of the training period.
The average active lever presses for saccharine at the beginning of the training period were 134.66 ± 14.94 and 168.22 ± 20.64 for group R and group V respectively which was statistically comparable to each other (Unpaired T test, $t(57)=-1.312$, $p=0.19$)).

Comparison of active lever presses at the beginning of the ethanol training period (Session 24-27)

The average active lever presses for ethanol were 45.69±2.82 and 51.61±3.96 for group R and group V respectively which was statistically comparable to each other (Unpaired T test, t(57)=-1.219, p=0.22)

Comparison of active lever presses in the middle of the ethanol training period (Session 44-47)

The average active lever presses for ethanol in the middle of the training period were 40.30±2.57 and 45.55±3.41 for group R and group V respectively which was statistically comparable (Unpaired T test, t(57)=-1.207,p=0.23).

Comparison of active lever presses towards the end of the ethanol training period (Session 77-80)

The average active lever presses for ethanol at the end of the training period were 31.49±2.06 and 48.04±2.61 for group R and group V respectively which was statistically significant (Unpaired T test, t(57)=-4.813,p=<0.0001)

Therefore, we did not observe any correlation between the three addiction-like criteria and saccharine or ethanol drinking at the beginning of the training sessions. A positive correlation appeared to be significant with the late stage of alcohol conditioning, confirming in our model that, like in human pathology, addiction-like behavior is observed after protracted periods of alcohol intake.

DISCUSSION

The diagnosis of alcohol use disorder in humans according to the DSM 5 criteria is achieved by counting the total number of positive criteria met by the individual. The severity is defined by the number of positive criteria, namely mild (2 or 3 criteria), moderate (4 or 5 criteria) and severe (6 or more criteria) (APA, 2013). Similarly, we

ranked the rats for the three addiction-like criteria and considered those falling in the 66th to 99th percentile of the distribution as positive for that criterion. This allowed us to divide the rats in 4 different groups depending on the number of positive criteria. Approximately 12% of the study population belonged to the 3crit rats, and fulfilled the hallmarks of addiction. This shows that even if all the rats developed self-administration of alcohol only a small group eventually becomes addicted. Thus, a small proportion of rats have the propensity and vulnerability to develop uncontrolled conditioned responses to alcohol cues, and possibly addiction-like behavior similar to that seen in humans (Anthony *et al*., 1994). By highlighting the heterogeneity of animal responses to alcohol cues, this model should bring precision medicine to psychiatry (Insel, 2014) by better addressing the inter-individual vulnerability to lose control over alcohol intake (Swendsen and Le Moal, 2011). And as such, it most likely will provide a roadmap for future investigations depicting the cellular and molecular brain adaptations responsible for the slow but irremediable transition towards uncontrolled alcohol consumption.

One doubt that can be raised is the rationale for selecting the top 33 percentiles for defining the criteria. However, it has been reliably shown that arbitrarily changing the selection threshold to top 25th percentile or top 40th percentile does not change the selection of the addiction phenotype (Deroche-Gamonet & Piazza, 2014), and does not artificially restrict the fraction of 3-crit rats to10-15%. In agreement with this assertion, a recent set of experiments using the addiction criteria (set at the top 33 percentiles) for discriminating rats at risk of losing control over palatable food intake demonstrated that 3-crit rats in this particular study only represented 3 to 4 % of the sampling group (de Jong *et al*., 2013).

Bibliography

American Psychiatric Association (2013) Diagnostic and Statistical Manual of Mental Disorders (5th ed.) American Psychiatric Publishing, Arlington, VA.

Anthony, J. C., Warner, L. A., & Kessler, R. C. (1994) Comparative epidemiology of dependence on tobacco, alcohol, controlled substances, and inhalants: Basic findings

from the National Comorbidity Survey. *Experimental and Clinical Psychopharmacology, 2*(3), 244-268.

Dayas, C. V, Liu, X., Simms, J. A., & Weiss, F. (2007). Distinct patterns of neural activation associated with ethanol seeking: effects of naltrexone. *Biological Psychiatry*, *61*(8), 979–89.

de Jong J.W, Meijboom K.E, Vanderschuren L.J, & Adan RA. (2013) Low control over palatable food intake in rats is associated with habitual behavior and relapse vulnerability: individual differences. *PLoS One*. 8(9):e74645.

Deroche-Gamonet, V., & Piazza, P. V. (2014). Psychobiology of cocaine addiction: Contribution of a multi-symptomatic animal model of loss of control. *Neuropharmacology*, *76 Pt B*, 437–49.

Insel TR. (2014) The NIMH Research Domain Criteria (RDoC) Project: precision medicine for psychiatry. *Am J Psychiatry, 171*(4):395-397.

Kharbouche H., Steiner N., Morelato M., Staub C., Boutrel B., Mangin P., Sporkert F., & Augsburger M. (2010). Influence of ethanol dose and pigmentation on the incorporation of ethyl glucuronide into rat hair. *Alcohol*, *44*(6), 507-14.

Lenoir M, Serre F, Cantin L, Ahmed SH (2007) Intense sweetness surpasses cocaine reward. *PLoS One*, *2*(8):e698.

Roberts, A. J., Heyser, C. J., & Koob, G. F. (1999). Operant self-administration of sweetened versus unsweetened ethanol: effects on blood alcohol levels. *Alcoholism, Clinical and Experimental Research*, *23*(7), 1151–1157.

Swendsen, J., & Le Moal, M. (2011) Individual vulnerability to addiction. *Ann N Y Acad Sci.,* 1216:73-85.

Legends to supplementary figures

Supplementary figure 1:

Progression of conditioned responses for ethanol during self-administration training. Repeated measures ANOVA showed a significant decrease in the ethanol taking in the 0Crit rats ($F_{2,24}$ = 23.10, p<0.0001), and post hoc Bonferroni's analysis revealed that the ethanol taking was significantly lower in the 77th-80th sessions as compared to 24th-27th session (p<0.0001) and 44th-47th sessions(p=0.0005). Also, the ethanol taking was significantly lower in the 44th-47th sessions as compared to the 24th-27th

session(p=0.0036). Repeated measures ANOVA showed a stable ethanol taking behavior in all other rats (1Crit rats, $F_{2,13} = 1.32$, p=0.28; 2Crit rats, $F_{2,12} = 0.998$, p=0.38; 3Crit rats, $F_{2,6} = 0.107$, p=0.89). This gives a pointer that the 0Crit rats become less and less interested in ethanol consumption while others maintain their ethanol taking behavior.
*Significant compared to Session 24-27.

Supplementary figure 2:

Evolution of body weight and ethanol consumption during self-administration training.
Body weight in the four criteria rats at the beginning (A) and at the end (B) of ethanol self-administration sessions. Ethanol intake expressed in g/kg in the four criteria rats at the beginning (C) and at the end of ethanol self-administration sessions (D). A one-way ANOVA showed comparable body weight across all the four groups at the beginning ($F_{3,55}$=2.054, p=0.17). and at the end of the experimental procedure ($F_{3,55}$=0.421, p=0.73). One way ANOVA revealed similar ethanol intake in rats at the beginning ($F_{3,55}$=0.508, p=0.69), but statistically significant difference towards the end of the experimental procedure ($F_{3,55}$=8.63, p<0.0001). A post-hoc Bonferroni's analysis showed that the 2Crit (p<0.0001) and 3Crit (p=0.0003) rats had significantly higher ethanol intake as compared to 0Crit rats.
*Significant as compared to 0crit.

Supplementary figure 3:

Daily results during evaluation of addiction-like criteria.
Here are presented the three consecutive sessions that have been averaged and summarized in Figure 1. Further statistical analysis is presented in the result section of this supplementary information.

Supplementary figure 1:

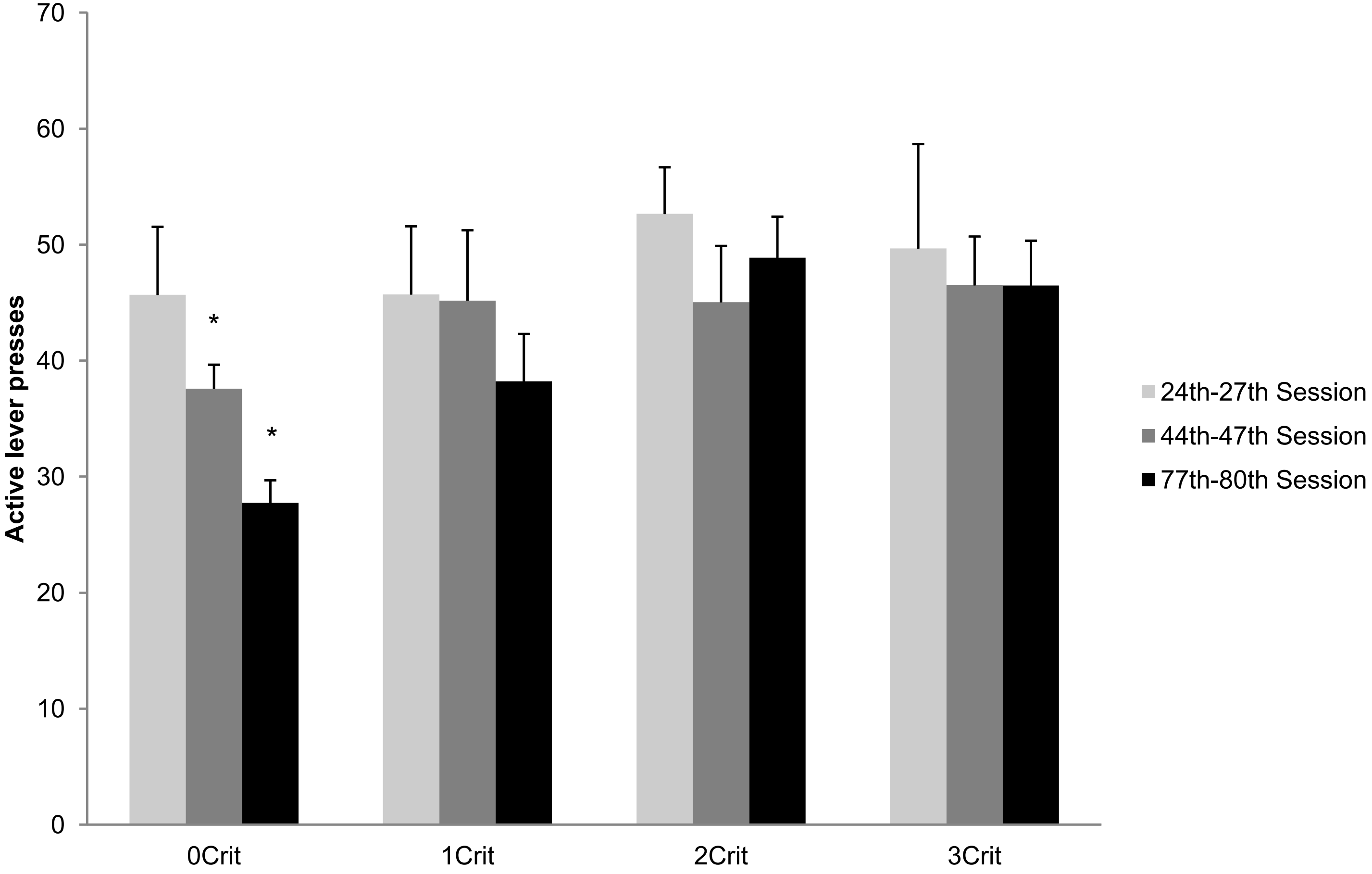

Supplementary figure 2:

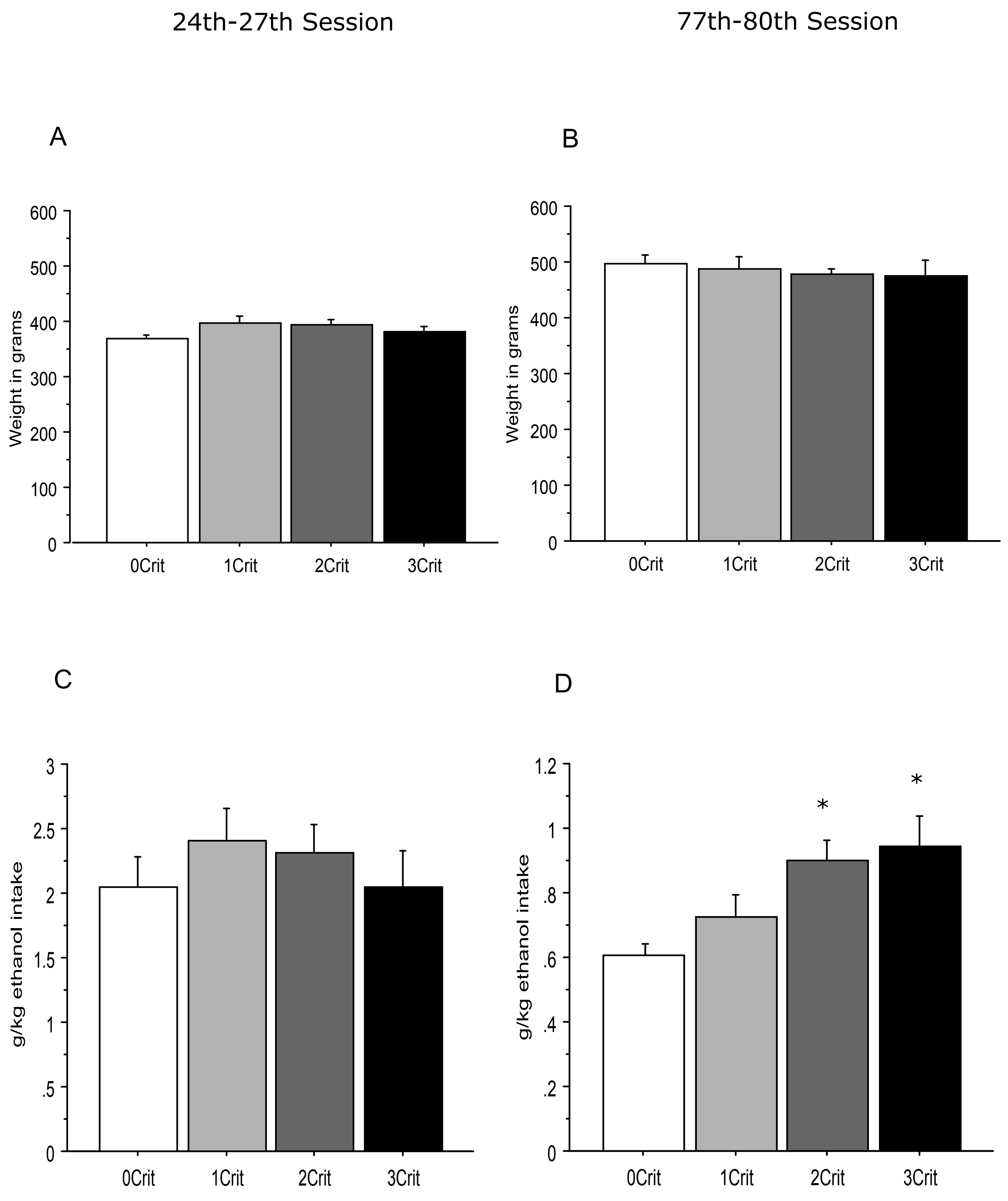

Supplementary figure 3:

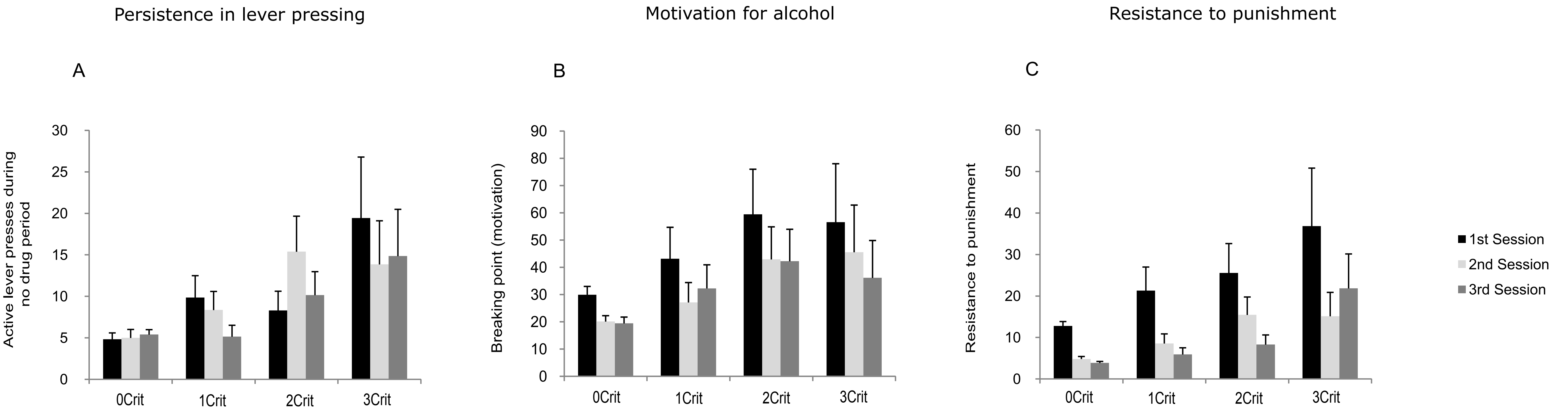

Supplement: Supplementary file 1 — Supplementary Information [file 41598_2017_9801_MOESM1_ESM.pdf]
